# Rhinitis, sinusitis and ocular disease – 2095. Inhibitory effect of Ginsenoside Rg1 on extracellular matix production via extracellular signal-regulated protein Kinase/ activator Protein 1 pathway in nasal polyp-derived fibroblasts

**DOI:** 10.1186/1939-4551-6-S1-P172

**Published:** 2013-04-23

**Authors:** Sung-Moon Hong, Heung-Man Lee

**Affiliations:** 1 Otorhinolaryngology-Head and Neck Surgery, Korea University Hospital, Seoul, South Korea; 2 Otorhinolaryngology-Head and Neck Surgery, Korea University College of Medicine, Guro Hospital, Seoul, South Korea

## Background

Nasal polyps are associated with chronic inflammation of the sinonasal mucosa and are involved in myofibroblast differentiation and extracellular matrix (ECM) accumulation. Ginsenoside Rg1, a compound derived from *Panax ginseng*, shows antifibrotic and anticancer effects. However, the molecular effects of Rg1 on myofibroblast differentiation and ECM production remain unknown. The aims of this study were to investigate the effect of Rg1 on transforming growth factor (TGF)-*β*1-induced myofibroblast differentiation and ECM production and to determine the molecular mechanism of Rg1 in nasal polyp-derived fibroblasts (NPDFs).

## Methods

NPDFs were isolated from nasal polyps of seven patients who had chronic rhinosinusitis with nasal polyp. NPDFs were exposed to TGF-*β*1 with or without Rg1. Expression levels of *α*-smooth muscle actin (SMA), fibronectin and collagen type I*α*1 were determined by reverse transcription polymerase chain reaction, Western blot and immunofluorescent staining. TGF-*β*1 signaling molecules, including Smad2/3, extracellular signal-regulated protein kinase (ERK), c-Jun N-terminal kinase (JNK) and p38 were analyzed by Western blotting. Transcription factors involved with TGF-*β*1 signaling, nuclear factor (NF)-*κ*B and activator protein 1 (AP-1) were also assessed by Western blot. The cytotoxic effect of Rg1 was measured by an established viability assay.

## Results

The mRNA and protein expression levels of *α*-SMA, fibronectin and collagen type I*α*1 were increased in TGF-*β*1-induced NPDFs. Rg1 inhibited these effects. The inhibitory molecular mechanism of Rg1 was involved in the ERK pathway. Rg1 inhibited the transcription factor activation of AP-1. Rg1 itself was not cytotoxic. The ginsenoside Rg1 has inhibitory effects on myofibroblast differentiation and ECM production. The inhibitory mechanism of Rg1 is involved with the ERK and AP-1 signaling pathways.

## Conclusions

Rg1 may be useful as an inhibitor of ECM deposition, and has potential to be used as a novel treatment option for nasal polyps.

